# The Cambridge Mental Health Film Club: lessons to learn, feedback, expansion and development of a standard operating protocol

**DOI:** 10.1192/bjo.2021.378

**Published:** 2021-06-18

**Authors:** Felix Clay, Emanuele Osimo, Sara Al-Nakeeb

**Affiliations:** Cambridge and Peterborough Foundation Trust

## Abstract

**Aims:**

To report on our progress and feedback running the Cambridge Mental Health Film Club. To share the recent development of a Standard Operating Protocol to help others organise Mental Health Film Screenings in order to foster discussion, engage the public, reduce stigma about mental health and build understanding.

**Background:**

Cinema lends itself to exploring social and mental health issues such as stigma in an enjoyable way within a limited time and budget. Viewing a film with those from different backgrounds and having a chance to discuss perspectives on meaning and significance is an effective way to promote a collaborative stance and expand perspectives. We have been running a Mental Health Film Club in Cambridge for the past 3 years and have recently celebrated our 10th screening.

**Method:**

We give details of our screenings and feedback. We also share our Standard Operating Protocol which covers important topics such as resources to find suitable films, obtaining copyright permission, finding suitable venues, supporting open discussions, use of social media and promoting inclusivity.

**Result:**

Our Mental Health Film club shows three films a year and over time has opened up to both professionals and members of the public who are interested in discussing mental health through movies and supporting recovery. We have screened many challenging and interesting films: from the impact of religious control on emergent adolescent sexuality (‘The Miseducation of Cameron Post’) to a classic film on Alcohol Dependency (‘Days of Wine and Roses’). We also support local festivals with a similar mental health theme (e.g. MEDFEST) and have recently run a very successful screening with the University of Cambridge Psychiatry Society which was introduced by a student offering subjective experience of growing up with a sibling with an Autism Spectrum Disorder (‘Life, Animated’). We promote screenings and publish all film discussions on our website (www.tinyurl.com/psychfilmclub) and Twitter in order to contribute to resources for educational use within Psychiatry training and to further involve the wider public. Feedback shows that our sessions are highly rated at helping audiences see mental health in a new way with post film discussion especially valued.

**Conclusion:**

Our experience and practical advice can inspire others to start a Mental Health Film Club and promote cohesion, resilience and collaborative thinking within their locality. For future events we plan to expand into more public engagement via local Film Festivals. We welcome delegates ideas, experiences and film recommendations.

